# New Insights on the Role of Manganese in Alzheimer’s Disease and Parkinson’s Disease

**DOI:** 10.3390/ijerph16193546

**Published:** 2019-09-22

**Authors:** Airton Cunha Martins, Patricia Morcillo, Omamuyovwi Meashack Ijomone, Vivek Venkataramani, Fiona Edith Harrison, Eunsook Lee, Aaron Blaine Bowman, Michael Aschner

**Affiliations:** 1 Department of Molecular Pharmacology, Albert Einstein College of Medicine, Bronx, NY 10461, USApatricia.morcillo@einstein.yu.edu (P.M.); 2 Department of Human Anatomy, School of Health and Health Technology, Federal University of Technology Akure, Akure 340252, Nigeria; godmamus@gmail.com; 3 Department of Hematology and Medical Oncology and Institute of Pathology, University Medical Center Göttingen (UMG), 37075 Göttingen, Germany; ramani@med.uni-goettingen.de; 4 Department of Medicine, Vanderbilt University Medical Center, Nashville, TN 37232, USA; fiona.harrison@Vanderbilt.Edu; 5 Department of Pharmaceutical Sciences, Florida A&M University, Tallahassee, FL 32301, USA; eunsook.lee@famu.edu; 6 School of Health Sciences, Purdue University, West Lafayette, IN 47907-2051, USA; bowma117@purdue.edu

**Keywords:** manganese, Alzheimer’s disease, Parkinson’s disease, neurodegeneration

## Abstract

Manganese (Mn) is an essential trace element that is naturally found in the environment and is necessary as a cofactor for many enzymes and is important in several physiological processes that support development, growth, and neuronal function. However, overexposure to Mn may induce neurotoxicity and may contribute to the development of Alzheimer’s disease (AD) and Parkinson’s disease (PD). The present review aims to provide new insights into the involvement of Mn in the etiology of AD and PD. Here, we discuss the critical role of Mn in the etiology of these disorders and provide a summary of the proposed mechanisms underlying Mn-induced neurodegeneration. In addition, we review some new therapy options for AD and PD related to Mn overload.

## 1. Introduction

Transition metals such as manganese (Mn), iron (Fe), cobalt (Co), and zinc (Zn) are essential for all life forms as at least 40% of enzymes require a metal as a constituent of many metalloenzymes serving as an enzyme activator [1,2]. As a naturally occurring mineral, Mn is one of the most abundant metals in the tissues of mammals and has been shown to function in many key biological processes, serving as a catalyst, enzyme cofactor, and gene modulator. Mn is required for a variety of physiological processes including brain and skeletal development, blood clotting, reproduction, neuronal function, antioxidant defense, maintaining immune integrity, and is, as recently revealed, also critically involved in anti-viral innate immunity [3,4,5]. Mn is found in soil, water, and food legumes and has 11 oxidation states, but it is predominantly in biological tissues as Mn^2+^ and Mn^3+^ [6,7]. Mn deficiency has been associated with increased susceptibility to seizures, birth, and skeletal defects [7,8,9]. 

Normal Mn concentration varies depending on the biological tissues evaluated. Generally, the estimated value of Mn in the body is about 4–12 μg/L in whole blood, 1–8 μg/L in urine, and 0.4–0.85 μg/L in serum [10,11]. Industrial sources of exposure are a major public health challenge. The inhalation of Mn particulates may occur during occupational activities (such as mining and welding) or the use of Mn containing materials during production [12,13,14,15]. Another pathway of exposure is the use of total parenteral nutrition (TPN) to manage health conditions such as gastrointestinal tract and liver damage [16]. Individuals receiving long-term TPN are at risk of suffering from extrapyramidal motor dysfunction associated with Mn accumulation in various brain regions [17]. Moreover, experimental investigations with a Mn enhanced diet in developing rats have demonstrated increased Mn accumulation in certain brain regions and alterations in brain neurotransmitters [18].

A multitude of neurodegenerative disorders such as Parkinson’s disease (PD), Alzheimer’s disease (AD), Huntington’s disease (HD), and amyotrophic lateral sclerosis (ALS) are characterized by metal dyshomeostasis, which may also contribute to the disease-specific protein aggregations [19,20].

Studies are beginning to show links between brain Mn concentration and the development of AD or AD-like symptoms, although the data are mixed as to the nature of the relationship. Tong et al. [21] showed increases in plasma Aβ peptides (one of the two key proteins implicated in AD) associated with an increased concentration of Mn. This was presumed to indicate a greater Aβ burden in the brain. In AD, the Aβ peptide is proteolytically cleaved from the larger amyloid precursor protein (APP) at a higher rate than normal. In contrast, exposure of neuroblastoma cell line SH-SY5Y cells to Mn led to decreased viability and lower expression of APP, which could also limit non-amyloidic cleavage to protective sAPPα [22,23]. Mn exposure also altered the ratio between the Fe^2+^ and Fe^3+^ driving oxidative environment, which may also drive APP toward amyloidogenic cleavage in the human brain, although this was not tested directly [23,24]. However, a meta-analysis of 17 studies showed an association between decreased serum Mn levels of AD and patients with mild cognitive impairment (MCI), suggesting a potentially contributory role for Mn deficiency in disease development [25]. 

Excess accumulation of Mn is called manganism. This disorder has phenotypic features analogous to idiopathic PD with some distinct differences [26]. Mn-induced parkinsonism has been shown to affect dopaminergic neurons as well as other monoaminergic neurotransmitters [3,27] predominantly in the basal ganglia (including striatum, globus pallidus, and substantia nigra) and limbic structures [28,29]. Table 1 summarizes the main findings in PD, parkinsonism and AD regarding the motor and non-motor symptoms, affected areas, pathologic phenotype, and pathophysiological mechanisms.

Given the above, this review addresses the involvement of Mn in cellular and molecular mechanisms that cause neurodegeneration and the accompanying symptoms inherent to AD and PD.

## 2. Oxidative Stress and Mitochondrial Dysfunction

Oxidative stress plays important roles in neurodegenerative disorders such as PD and AD and its progression through an imbalance between reactive oxygen and nitrogen species production and neutralization by endogenous antioxidant defense mechanisms [30]. Due to the high metabolic activity of neurons consuming at least 20% of the total oxygen and calories, the brain is highly vulnerable to oxidative injury [31,32]. The generation of unstable molecules such as superoxide radical (O_2_^•^^−^), hydrogen peroxide (H_2_O_2_), and hydroxyl radical (^•^OH) that can interact with biological macromolecules, leads to structural changes and damage in lipids, proteins, and nucleic acids. 

Studies have shown that Mn is involved in significant changes in the level of activity of the antioxidant enzymes including isoforms of superoxide dismutase (SOD), catalase (CAT), and glutathione peroxidase (GPx) [33,34]. Furthermore, Mn contributes to oxidative stress by increasing production of nitric oxide through the activation of the inducible nitric oxide synthase in astrocytes [35]. Indeed, astrocytes have been linked to neurotoxic effects by the activation of proinflammatory responses [36]. In this regard, earlier studies have reported that after Mn exposure, NF-κB in astrocytes may stimulate the production of inflammatory cytokines and chemokines (including IL-6, TNF, CCL2, and CCL5) [37,38,39] Moreover, it have been demonstrated that Mn exposure increases mRNA expression of pro-inflammatory cytokines, IL-1b, TNF-α, and COX-2. These studies suggest that the astrocytic NF-kB pathway may have a critical role in inflammatory signaling processes in neurodegenerative diseases induced by Mn [38,40]. On the other hand, excessive Mn exposure can directly evoke free radical formation due to the redox-active nature of this transition metal, resulting in direct oxidation of membrane lipids, DNA, amino acids, neurotransmitters, and other relevant biomolecules [41]. Regarding neurodegenerative diseases, Balmus et al. [42] recently demonstrated that high Mn levels in AD patients were strongly positively correlated with low antioxidant defenses, measured as decreased GPx activity, and increased lipid peroxidation measured as enhanced malondialdehyde (MDA), secondary to excessive ROS production. In addition, alternative and complementary animal models used in the study of PD such as *Caenorhabditis elegans* and *Drosophila melanogaster* have confirmed a relationship between Mn exposure and changes in oxidative parameters such as decreased glutathione levels, enhanced MDA (that measure of lipid peroxidation) as well as levels of protein carbonyls (a measure of protein oxidation) [43].

Mechanistically, it has been proposed that Mn-induced oxidative stress in neurodegenerative diseases might be secondary to excessive iron (Fe) accumulation. Mn has been shown to block protein translation of APP, which is responsible for the stabilization of the membrane-bound Fe^2+^-exporter ferroportin and heavy-chain Ferritin (H-Ferritin). These proteins sequester Fe^2+^ via conversion to redox inactive Fe^3+^ by increasing the binding of iron regulatory protein-1(IRP1) to the iron responsive element (IRE) on the 5’-UTR of APP and H-Ferritin mRNA [23,44]. This is important since Fe^2+^ is a major intrinsic generator of ROS, responsible for the decomposition of H_2_O_2_, and in turn, producing OH hydroxyl group by the Fenton reaction and Haber-Weiss, thereby contributing to Mn-induced oxidative stress [45,46].

Mn-induced oxidative stress in neurodegenerative diseases can also be secondary to a mitochondrial dysfunction, which plays a central role in PD and AD [47,48,49]. Mn^2+^ interferes with Ca^2+^ homeostasis within the mitochondria by occupying Ca^2+^ binding sites [50,51], triggering an increase in mitochondrial Ca^2+^ levels, which interfere with oxidative respiration and induce oxidative stress [52]. The ROS generated by excessive Mn levels promote the opening of the mitochondrial permeability transition pore, causing a loss of membrane potential and impairing ATP synthesis and mitochondrial swelling, thereby contributing to cellular apoptosis [53,54]). Moreover, Mn may directly affect mitochondrial homeostasis by inhibiting the electron transport chain, leading to decreased ATP formation, increased leakage of electrons, and enhanced O_2_^•−^ generation [55]. Indeed, Mn inhibits electron transport chain in two independent sites in brain mitochondria: while the primary site is electron transport chain complex II [56,57,58], the inhibition of complex I activity in isolated rat brain mitochondria has also been reported [50,59] or in mesencephalic DA neurons derived from human induced pluripotent stem cells (hiPSC) [60].

Taken together, these findings suggest that Mn promotes neurotoxicity associated with AD and PD by fueling ROS production, causing cellular oxidative stress and perturbing the mitochondrial electron transport chain impairing mitochondrial enzyme activity and mitochondrial membrane potential affecting ATP-dependent energy production.

## 3. Mn-Mediated Regulation of Amyloid Precursor Protein (APP) and Amyloid-β (Aβ) Aggregation

Aside from aggregates of the hyperphosphorylated tau protein building neurofibrillary tangles, the formation and deposition of Aβ plaques is a cardinal feature of AD pathogenesis [61]. Aβ plaques are produced by sequential cleavage of APP via α-, β-, and γ-secretase enzymes to produce APP fragments including Aβ and c-terminal fragments. The majority of APP cleavage is via α-secretase, which cleaves APP between amino acids 612 and 613 and produces soluble APPα, which possesses neuroprotective features. In contrast, amyloidogenic cleavage occurs when APP is cleaved by β- and γ-secretases generating Aβ monomers. These monomers aggregate to generate oligomers and fibrils, which are considered to be neurotoxic. Ultimately, continued aggregation of Aβ peptides into β-sheets generates the hallmark senile plaques [62,63]. A recent study showed the binding of Mn to the monomeric Aβ peptide in vitro, without any major effects on Aβ aggregation and no obvious effects on plaque formation. These data suggest weak and transient interactions in this binding, since aggregation kinetics and fibril morphology of Aβ do not change according to the presence or absence of Mn when monitored by spectroscopy and fluorescence experiments [64]. Moreover, other metals such as copper (Cu) and zinc (Zn) found in the human brain have higher interaction with Aβ aggregation than Mn [65,66]. Therefore, additional studies are required to better understand the effects of Mn/Aβ interactions on Aβ aggregation and the molecular mechanisms involved [64,67]. 

Altered serum Mn level between AD and control populations has been observed in multiple studies. However, methodological limitations and a lack of consistency between study approaches have failed to accurately elucidate the role of Mn in AD [21,25,68,69,70]. High pollution in Mexico City led to selective accumulation of Mn (and not iron (Fe)) in brains, whereas the same changes were not observed in lung [71]. Mn accumulation in young adults was associated with diffuse Aβ plaques (in 51%) and hyperphosphorylated tau (in 40%), suggesting a direct impact on development of AD [72]. Similar pollution exposure led to increased expression of nuclear neuronal NF-κB and iNOS, altered blood–brain barrier (BBB) function, and diffuse Aβ plaques and neurofibrillary tangles in dogs [73,74]. Dogs represent a good model of pathological aging because they exhibit many of the same pathological signs of AD including Aβ accumulation and deposition. Intravenous Mn (3.3–5.0 mg/kg) in Cynomologous Macaques increased brain Mn independent of changes in Fe, upregulated expression of amyloid-β-like protein 1 mRNA (APLP1, a member of the amyloid precursor protein family), and led to diffuse Aβ plaques and degenerating cells [75,76]. These pathological signs were not observed in the control brains. Moreover, these Mn-treated groups showed impaired spatial working memory and fine motor skills, and increased compulsive like behaviors [76] each of which are associated with degenerative diseases and dementia. MnSOD expression in hippocampus was 3- to 11-fold greater in hippocampal regions CA1–CA3 in AD patients than the controls [77] tying differences in Mn handling to areas of the brain expressing high pathological change, and further supporting a direct relationship between an Mn-dependent antioxidant enzyme and Aβ. A similar neuropathology reported in canines, non-human primates, and humans strongly suggests a shared underlying pathway that is conserved across species. 

## 4. Aggregation of α-Synuclein in Mn-Induced Neurotoxicity

Alpha-synuclein (αSyn) is a chaperon protein with 140 amino acids widely expressed in neural tissue where it predominantly localizes to the presynaptic terminal. It can play important roles in the regulation of synaptic plasticity, vesicle transport, and dopaminergic neurotransmission [78]. αSyn is a protein with a natural tendency to aggregate into oligomers and is a key player in the pathology of PD [79,80]. In the same way, although nuclear magnetic resonance (NMR) studies have shown that αSyn has a poor affinity for Mn^2+^ in its C-terminal binding site, Mn^2+^ can trigger misfolding and accumulation of αSyn protein [81,82]. Indeed, emerging evidence indicates that αSyn oligomerization is a major culprit for Mn-induced neurotoxicity [83,84,85]. Further evidence that Mn regulates exosome-mediated extracellular micro RNA (miRNAs) comes from the MN9D dopaminergic cell culture model [86]. Mn exposure significantly upregulated the release of exosomes from cells to the extracellular environment in wild-type human αSyn-expressing MN9D dopaminergic cells. Moreover, Mn-induced exosomes contain miRNAs, which are involved in the regulation of key biological pathways including protein aggregation, autophagy, inflammation, and neurodegenerative disease [87,88]. Harischandra et al. [89] demonstrated that Mn also promotes the aggregation and prion-like exosomal transmission of αSyn from cell to cell, resulting in dopaminergic neurotoxicity in a mouse model of Mn^2+^ exposure. Together, these results indicate that Mn^2+^ exposure promotes αSyn secretion in exosomal vesicles, which subsequently evokes proinflammatory and neurodegenerative responses in both cell culture and animal models. 

Mn exposure may induce neurotoxicity by the overexpression of αSyn, leading to enhanced αSyn levels and resulting in αSyn aggregation and misfolding [84,90]. Indeed, αSyn oligomerization might be the major factor responsible for Mn-induced autophagy dysregulation and neuronal injury [91]. Such a role is plausible given the requirement for autophagic degradation of αSyn monomers or oligomers [92] and the role of dysregulated autophagy in the development of neurodegenerative disorders [93]. In α-Syn knockout mice (α-Syn^−/−^) excessive autophagy and aggravation of apoptosis following Mn exposure compared to control mice has recently been shown [91]. These results support the potential neuroprotective role of αSyn in ameliorating Mn-induced excessive autophagy and neuronal injury.

## 5. Mn-Mediated Effects on the Cholinergic System

The cholinergic system encompasses the neurotransmitter acetylecholine (ACh), that is synthesized from choline and acetyl-coenzyme A, mediated by choline acetyltransferese (ChaT) and stored in the presynaptic vesicles. Once released into the synaptic cleft, ACh binds to pre- and postsynaptic muscarinic and nicotinic receptors and is hydrolyzed by the enzymes cholinesterases (acetylcholinesterase (AChE) and butyrylcholinesterase (BuChE)). The cholinergic system plays an important role in cognitive domains involved in learning, attention, and memory [94,95]. In fact, impairments in the cholinergic system are associated with several illness such as myasthenia gravis and AD [96]. In AD patients, loss of neurons in the basal forebrain leads to dramatic changes in cholinergic innervation in the cortex and the hippocampus. This cell loss is correlated with memory and attention deficits [97,98,99]. Presynaptic cholinergic dysfunctions, over-activation of acetylcholinesterase (AChE), decrease in levels of ACh followed by dysfunction and eventual death of cholinergic neurons, support the cholinergic hypothesis of the disease [97].

Several studies have shown that Mn perturbs the cholinergic system, leading to locomotor, emotional, behavioral, and cognitive dysfunction. Mn can modify the activity of enzymes involved in cholinergic transmission such as AChE [100,101]. Rats treated with Mn in drinking water for 30 days showed significant increase in AChE activity as well as enhanced AChE expression in the cerebellum [100]. Similarly, short-term administration of MnCl_2_ enhanced AChE activity in rat brains [102]. Moreover, in a study with rats treated with a Mn enriched diet, brain extracts showed increased AChE activity compared to the control group [101]. In contrast, long-term treatment of rats with Mn in drinking water (approximately 40 mg Mn/kg-day) for over two years resulted in significant decreases of AChE activity in the hypothalamus, cerebellum, and striatum [103,104]. These conflicting results after Mn exposure on AChE activity suggest that the effects of Mn may depend on age, dose, route of exposure, frequency, and duration [81,101,105].

Mn-induced inhibition of AChE activity in the rat brain increased F2-isoprostanes levels in a dose-dependent manner, suggesting that Mn promotes changes in neuronal oxidative stress and neuroinflammatory biomarkers, possibly due to the inhibition of AChE [106,107]. Mn exposure via intranasal administration resulted in increased oxidative stress and reduced nicotinic acetylcholine receptor levels in the prefrontal cortex, suggesting that Mn contributes to cholinergic neurotransmission disruption in the brain areas critical for cognition [108]. Mn also induced cholinergic neurodegeneration in *C. elegans* including upregulation of mRNA of *ace-2*, an enzyme responsible for hydrolyzing ACh into acetate and choline. These changes suggest upregulation of cholinergic degradation enzyme, which can lead to impaired behavioral parameters such as pharyngeal pumping and body bends for analysis of locomotion [109]. In agreement with these findings, studies in vitro reported increased AChE activity in cultured cells due to oxidative stress [110]. Moreover, studies suggested that AChE activity has been demonstrated to increase Aβ formation within and around amyloid plaques and makes clear the involvement of this system in the pathogenic development of AD by influencing the process that leads to amyloid toxicity [111]. Given the role of Mn in mediating cholinergic function, and the links between cholinergic changes including AChE activity, Aβ generation and aggregation, and oxidative damage in AD, it is clear that there is a strong potential role for Mn in AD disease development.

## 6. Mn-Induced Parkinsonism and the Involvement of the Dopaminergic System

The neurotransmitter system that has received the most attention in the study of Mn neurotoxicity is the dopaminergic (DAergic) system. The neurobiological basis for the effect of Mn on dopamine metabolism, neurotransmission, and selective accumulation of Mn in the basal ganglia following overexposure has yet to be fully delineated [7]. Several studies have shown that the divalent metal transporter 1 (DMT1), an important transporter of Mn (and other divalent ions) in the brain, is highly expressed in the basal ganglia. DMT1 upregulation has been posited to be associated with oxidative stress and dopaminergic cell loss, suggesting this transporter may contribute to neurodegeneration [112,113,114]. Moreover, it is clear that Mn overexposure induces DAergic neurodegeneration [115,116,117,118]. This result is supported by neuroimaging modalities such as positron emission tomography (PET), single-photon emission computed tomography (SPECT), and magnetic resonance imaging (MRI) [7,119]. In PD, both PET and SPECT imaging showed altered dopamine neuron function and terminal density in key pathological areas such as the dorsal striatum, but preserved or even increased postsynaptic D2 dopamine receptor raclopride binding (D2R) [120,121]. In contrast, Mn-exposed workers, nonhuman primates, or rodents, revealed normal flurodopa uptake and dopamine transporter (DAT) density, decreased dopamine release, and D2R in the striatum [14,118], and no decrease in the numbers of substantia nigra pars compacta neurons [116,122]. A recent report in non-human primates provides evidence of Mn-induced alterations in PET imaging of the frontal cortex DAR and D1-dopamine receptor (D1R) that may be associated with working memory and attention deficits observed in Mn-exposed subjects [123]. Conversely, using a new technique called nondisplaceable binding potential (NMB) PET, nigral D2R increased in workers and welders with Mn exposure and clinical parkinsonism, indicating dose-dependent dopaminergic dysfunction of the SN secondary to Mn exposure [124]. Increases in striatal D2R binding may represent compensatory upregulation of striatal D2Rs in early stages of PD, referred to as D2R denervation supersensitivity [125]. However, with progressive degeneration of nigrostriatal pathways, striatal D2R binding decreased to normal or reduced levels when compared to healthy controls [126,127].

Studies using cell cultures consistently demonstrate that DA neurons are susceptible to Mn exposure [128,129,130], however, the exact cellular and molecular mechanisms of Mn-induced neurotoxicity remain elusive. Mn-induced neurotoxicity in dopaminergic neurons has been studied, and several key players such as protein kinase C delta (PKCd) (131) and caspase 3 signaling have been identified [131,132]. Furthermore, Mn exposure induces mitochondria-mediated apoptosis in neurons, which is partially facilitated by p53 [133], and DNp73 antagonizes the functional p53 by regulating the expression of anti-apoptotic molecules such as Bcl-xL and Mcl-1 [134]. In addition, p73 gene expression resulting in enhanced susceptibility to apoptotic cell death in N27 dopaminergic neuronal mode [135]. c-RET has been reported to be associated with the dopamine-producing pathway through enhancing the transcription of tyrosine hydroxylase (TH), which is a rate-limiting enzyme in dopamine biosynthesis [136]. TH has been used as one of the indicators to determine the level of dopamine production [137]. 

## 7. Mn-Mediated Effects on the GABAergic System

Excessive exposure to Mn can cause a variety of effects not only in the striatum, but also in the globus pallidus [138] as well as abnormalities in GABAergic transmission [139]. Indeed, Mn accumulates in the basal ganglia, particularly in the globus pallidus [140]. GABA plays a key role in mediating the direct and indirect pathway of the basal ganglia, both of which have GABAergic projections to the thalamus [141]. The basal ganglia-thalamocortical pathway is mainly involved in the regulation of motor coordination [142]. In this context, studies have evaluated the effects of Mn on GABA and controversial results have been found [7]. Studies on non-human primates have shown no change in brain GABA concentration under Mn exposure [27,143]. In contrast, recent investigations using magnetic resonance spectroscopy have found elevated thalamic GABA levels in lower-exposure occupational setting [144,145] and PD patients [146]. In addition, Ma et al. [138] have shown with edited magnetic resonance spectroscopy and MRI an increase of thalamic GABA levels in a group of welders with higher exposure to Mn as well as poorer performance in general motor function. However, welders with lower Mn exposure did not differ from the controls in GABA levels or motor performance. Furthermore, in welders, the thalamic GABA levels were best predicted by past-12-months of Mn exposure levels and were influenced by the Mn deposition in the substantia nigra and globus pallidus. Importantly, both thalamic GABA levels and motor function displayed a non-linear pattern of response to Mn exposure, suggesting a threshold effect. These data suggest that the effects of Mn on the GABAergic system are complex. However, results on striatal GABA concentrations in PD are controversial [146,147]. Accordingly, Casjens et al. [148] did not provide evidence that striatal and thalamic GABA differ between Mn-exposed workers, PD patients, and controls. Differences in species, length of exposure duration, and the challenge to detect small changes in GABA could all play a role in the effect of Mn neurotoxicity. Therefore, more research is necessary to establish the effect of Mn exposure on GABA neurotransmission.

## 8. New Therapies to Treat Mn-Induced Parkinsonism and AD

Although different therapeutic approaches have been studied in Mn-induced neurotoxicity [7,81], there is currently no protective strategy against Mn neurotoxicity. Originally, patients with Mn-induced parkinsonism were treated with levodopa, but were unresponsive to the treatment, possibly due to the relatively intact nigrostriatal pathway in the latter phase of the disorder [149,150]. More recently, mitochondria have been highlighted as a therapeutic target against Mn neurotoxicity. In animal models, the amino acid taurine has been investigated as a potential treatment for manganism. In mice, taurine alleviated Mn-induced locomotor deficits, mitigated oxidative stress biomarkers, and preserved indices of mitochondrial functionality in brain tissue [151]. Additionally, taurine administration preserved mitochondrial ATP, prevented mitochondrial depolarization and swelling, and increased mitochondrial dehydrogenases activity [152]. Interestingly, it has also been found that Mn neurotoxicity is associated with disturbances in taurine homeostasis [153]. Furthermore, co-administration of taurine improves the spatial learning and memory ability impaired by sub-chronic Mn exposure [154]. Other treatment options include rasagiline, a monoamine oxidase inhibitor that inhibits the metabolism of striatal dopamine used in the clinic for PD. Ragasaline provided a small, but significant protection against the initial Mn-induced reactive oxygen/nitrogen species (RONS) formation as measured in iPSC- derived human dopamine neurons [60]. 

Recently, the use of bioinformatic methods has been trialed to predict the possible molecular mechanisms underlying Mn-induced AD and screen possible molecules to reverse the neurotoxicity or AD development. Using the connectivity map (CMAP) tool, it was demonstrated that Tyrphostin AG-825, an inhibitor of tyrosine phosphorylation, could be a potential agent for overcoming Mn-induced neurotoxicity or AD development [155]. This molecule is specifically inhibiting ErbB2 (a member of the epidermal growth factor receptor (EGFR)/ErbB family) that plays an important role in the pathogenesis of AD and is strongly associated with neuritic plaques in AD [156], suggesting that this molecule could be used in future studies to reverse the biological process related with neurotoxicity induced by metal.

## 9. Conclusions

Although Mn has an important role in physiological functions, especially in the brain, overexposure to this metal leads to toxic effects. Studies have shown that oxidative stress and the imbalance in mitochondrial energy metabolism are involved in Mn neurotoxicity, which induces or mediates AD and PD. Moreover, neurotransmitter systems such as the cholinergic system, dopaminergic system, and GABA system might be affected by Mn, suggesting the involvement of these neurotransmitter systems in these neurological diseases. Overall, the findings summarized in this review warrant further investigation into the molecular mechanisms and pathophysiological interrelationship between Mn exposure and neurodegenerative disorders such as AD and PD.

Moreover, new approaches to validate novel biomarkers and create better disease models to AD and PD induced by Mn as well as proteomic and transcriptomic analysis alongside novel bioinformatic tools are essential for advancing knowledge of the disease mechanisms induced by Mn. Furthermore, studies to investigate potential molecular targets and new therapeutic strategies for these devastating illnesses are urgently required.

## Figures and Tables

**Table 1:** An overview of the different findings in PD, parkinsonism, and AD.

Parameter	Parkinson Disease	Parkinsonism	Alzheimer’s Disease
Motor symptoms	- Rigidity - Bradykinesia - Resting tremors	- Rigidity - Bradykinesia - Steppage gait - Less tremors - Painful limb spasms - Dystonia	- Not common - Eventually walking difficult - Swallowing difficulties
Non motor symptoms	- Depression - REM sleep behavior disorders	- Dementia - Hallucinations - Memory loss - Disorientation - Illusions/Delusions	- Cognitive impairment - Dementia - Memory loss
Affected areas	- Substantia nigra, critical for dopamine synthesis	- Mainly in basal ganglia, - Cerebellum, red nucleus, cortex, thalamus and anterior horn of the spinal cord	- Loss of neurons in the cortex and hippocampus
Pathologic phenotype	- Dopaminergic neurons degeneration - Lewy bodies - Therapeutic response to levodopa - Fluorodopa uptake	- Nigrostriatal dopaminergic dysfunction - Absence response to levodopa - Failure to detect fluorodopa uptake	- Neurofibrillary tau tangles - Amyloid Aβ plaques - cell loss - brain shrinkage
Pathophysiological mechanisms	Oxidative stress, protein aggregation, impaired proteasomal and autophagy functions, excitotoxicity, aberrant signal transduction, mitochondrial dysfunction and cell death pathways.		- Oxidative stress, mitochondrial dysfunction, decrease in cholinergic innervation, neuroinflammation
